# Currently recommended skin scores correlate highly in the assessment of patients with Juvenile Dermatomyositis (JDM)

**DOI:** 10.1186/s12969-023-00844-5

**Published:** 2023-06-28

**Authors:** Alexander Gebreamlak, Katherine M. Sawicka, Rose Garrett, Y. Ingrid Goh, Kayla M. Baker, Brian M. Feldman

**Affiliations:** 1 Child Health Evaluative Sciences Program, SickKids Research Institute, Toronto, Canada; 2 Division of Epidemiology, Dalla Lana School of Public Health, University of Toronto, Toronto, Canada; 3 Institute of Health Policy, Management and Evaluation, Dalla Lana School of Public Health, University of Toronto, Toronto, Canada; 4 Department of Medicine, Division of Neurology, University of Toronto, Toronto, Canada; 5 Division of Biostatistics, Dalla Lana School of Public Health, University of Toronto, Toronto, Canada; 6 Department of Pediatrics, Division of Rheumatology, The Hospital for Sick Children, University of Toronto, 555 University Ave, 555 University Ave, Toronto, Canada

**Keywords:** Juvenile dermatomyositis (JDM), Disease activity score skin subscale (skinDAS), Cutaneous assessment tool (CAT), Cutaneous dermatomyositis disease area and severity index (CDASI), Physician’s global assessment skin visual analog scale (Skin VAS), Patient outcomes

## Abstract

**Background:**

Juvenile Dermatomyositis (JDM) is a rare, chronic, and life-threatening childhood autoimmune disease. Currently, there are recommended, reliable and validated measurement tools for assessment of skin disease activity in JDM including the Disease Activity Score (skinDAS), Cutaneous Assessment Tool (CAT), and the Cutaneous Dermatomyositis Disease Area and Severity Index (CDASI). The Physician’s global assessment skin visual analog scale (Skin VAS) is also widely used for skin activity in JDM. For the purpose of comparative international studies, we wanted to compare these tools to the Physician’s skin VAS (as a standard) to identify which performs better.

**Objectives:**

We sought to compare the correlations of these scoring tools, and separately assess the responsiveness each tool demonstrates following patient treatment, in order to see if one tool may be preferred. This was determined by assessing how well these tools correlate with each other, and the Physician’s skin VAS over time, as well as the responsiveness of each tool after patient treatment.

**Methods:**

Skin scores were recorded at a baseline (first visit after June 1^st^, 2018) and all follow-up office visits at the Juvenile Dermatomyositis Clinic. Following baseline visits, patients were followed up as clinically indicated. A subset of newly diagnosed patients (inception cohort) was identified. Correlations were assessed at the baseline visit and over time for the whole cohort. The correlations over time were derived using Generalized Estimating Equations (GEEs). Standardized response means with 95% confidence intervals were calculated to test score responsiveness for the nested inception cohort.

**Results:**

The skinDAS, CAT and CDASI all correlated highly with each other and with the Physician’s skin VAS. The three scoring tools accurately reflected Physician’s skin VAS scores over time. In addition, all tools showed moderate to high responsiveness following treatment.

**Conclusion:**

All studied skin score tools performed well in our study and appear to be useful. Since no tool far outperforms the others, arbitrary consensus will be needed to select a single standard measurement tool for the purposes of efficiency and global comparability.

## Introduction

Although childhood rheumatic diseases are currently not curable, they are treatable. The development of standardized outcome measures has been pivotal in assessing and following response to treatment.

Juvenile Dermatomyositis (JDM) is the most common chronic childhood inflammatory myopathy. It presents with skin rash and frequently with muscle inflammation [1]. JDM severity varies, and the disease affects all children differently; however, skin rash is often the first sign of disease and can be a marker of its progression [2].

Quantifiable outcome measures are fundamental for determining and tracking disease activity for JDM patients. Clearly defining and standardizing these measures aids in assessing response to treatment. Currently, there are several core data set measures and scoring assessment tools designed for the assessment of JDM disease activity. The International Myositis Outcome Assessment Collaborative Study Group (IMACS) has proposed a set of core set measures, and response criteria, based on international consensus, and a second consensus-driven data set, used with JDM patients, is from the Paediatric Rheumatology International Trials Organization (PRINTO) [3–5].

It has been suggested that the IMACS and PRINTO core sets both undervalue the importance of skin rash in JDM; although both core set measures include clinical assessment for cutaneous disease activity (judged by the Skin VAS), the current criteria are heavily weighted toward muscle disease [6, 7].

Quantifying skin disease, though, appears to have clinical utility. Studies demonstrate the importance of early and adequate treatment for JDM skin disease in preventing worse outcomes [8, 9]. JDM skin disease is often more recalcitrant to therapeutic interventions than muscle disease, and is often more prominent during the course of illness [8, 10]. Moreover, skin rash may be associated with poor long-term outcomes, i.e., calcinosis, poor quality of life and limited physical function [7, 11].

There are several measurement tools that are used to assess skin disease activity; these were developed with different goals, and different specificity of assessment, in mind.

Validated in 2003, the Disease Activity Score (DAS) assesses the extent and development of muscle weakness and cutaneous involvement with values ranging 0–20 [13]. This measurement tool quantifies a wide disease activity range for both the skin and muscle components while producing a reliable disease activity estimate [13]. Within the 20 point scale, 11 points are attributed to muscle disease with the remaining nine given for skin disease (skinDAS). The distribution and severity of the skin rash as well as the presence of vasculitis and Gottron papules are scored [12].

The Cutaneous Assessment Tool (CAT) was designed to grade skin activity and damage for juvenile idiopathic inflammatory myopathies[14]. Of the 21 items, the skin disease activity score uses 10 items, and the skin disease damage score uses four items, with an additional seven items common to both categories of the CAT [12]. Aside from lesion presence, the clinician performing the test also grades various lesion characteristics depending on severity [12].

The Cutaneous Dermatomyositis Disease Area and Severity Index (CDASI) was designed for both adults and children with dermatomyositis. This tool was developed to be a reliable and validated skin disease assessment tool to monitor skin disease progression longitudinally [15, 16]. This score examines 15 anatomical locations and activity scores range from 0–100 (with damage scores ranging from 0–32) [15]. After test completion by a clinician, disease activity levels are categorized as low, moderate or high, depending on respective cut-off values [12].

The Physician’s global skin assessment, as measured by a 10 cm visual analog scale (VAS), is widely used to quantify skin activity [12]. Physicians grade skin disease activity with higher values representing greater severity [12]. The Skin VAS is recommended at every visit in the international consensus dataset [17]. It is thought that the Skin VAS reflects the overall skin disease activity – as determined by the assessing physician – which incorporates all elements of the skin examination, and values these elements according to the physician’s judgement. As such, we felt that the Skin VAS was a good measure by which to judge the other, more stringently delineated, tools.

It is unclear if any of these tools offers advantages over the others. Past studies comparing construct validity, internal consistency and degree of responsiveness between the CDASI and CAT support the reliability and construct validity for both measurement tools when compared to the Skin VAS [16, 18]. Investigation of the validity for the CDASI and CAT found both to be significant predictors of the Skin VAS [18]. However, it was also seen that the CDASI displayed higher intra-rater reliability and greater responsiveness than the CAT with standardize response mean values > 1 [18]. In comparison, the DAS has demonstrated good validity relative to other indicators of disease outcomes [13]. It has also been shown to produce reliable measurements of single construct disease activity, but it may be insensitive at low levels of JDM [13]. Furthermore, the DAS, as a disease-specific global tool, has shown greater responsiveness for detecting clinically important change compared to some other core set measures [12].

All four tools are being utilized for collection into a number of ongoing disease registries. For the global community, however, we felt it was important to provide comparative information about these tools to further efforts into choosing a single tool for broad use. Thus, in this retrospective cohort study, our primary objective was to determine if there was an optimal tool for measuring JDM cutaneous disease – that is more responsive yet correlating highly with the other tools and with the physician’s judgement as per the Skin VAS, with the aim of reducing redundancy while maintaining high responsiveness.

We asked, i) In children under the age of 18 diagnosed with JDM, do the recommended measurement tools – the skin portion of the Disease Activity Score (skinDAS), Cutaneous Assessment Tool (CAT), and Cutaneous Dermatomyositis Disease Area and Severity Index (CDASI) – correlate highly with the Physician’s skin visual analog scale (Skin VAS) over time? ii) In *new-onset* JDM patients, who have received effective treatment, do the skinDAS, CAT, CDASI and Skin VAS show high responsiveness (as measured by the standardized response means (SRM) following the first three months of treatment? iii) Can any one of the skinDAS, CAT, CDASI (or even Skin VAS) be used, instead of all four, for optimal data collection in registries or in clinical practice?

## Methods

### Participant sampling

This study was approved by the Research Ethics Board (REB) at The Hospital for Sick Children (SickKids), Toronto, Ontario. The participant sample came from the Juvenile Dermatomyositis Clinic at SickKids. The total cohort was comprised of 77 participants; this included both existing and newly diagnosed patients (i.e., prevalent patients). We used the first visit following June 1^st^, 2018 as the baseline visit (that was the day that we started collecting all the skin scores as part of routine clinical practice). For the calculation of skin score responsiveness, we used a subgroup of 25 participants who had been newly diagnosed (and started on treatment) after June 1^st^, 2018.

The inclusion criteria were i) classified as probable or definite Juvenile Dermatomyositis according to the European League Against Rheumatism/American College of Rheumatology (EULAR/ACR) classification criteria [19], and ii) two visits to the JDM Clinic occurring within a 3-month period. Descriptive (demographic) data and disease variables were abstracted from standardized proforma, completed at each visit by trained clinicians, and recorded in the electronic medical record. We collected age, sex, diagnostic certainty (probability score as per the EULAR/ACR criteria) [19], physical strength and endurance (MMT-8/CMAS) [20, 21], functional ability (CHAQ) [22], disease severity at onset (baseline Physician’s skin VAS score), medications prescribed, and Myositis-Specific Antibodies (MSA, as tested by commercial immunoblot).

### Data collection

The skin scores (skinDAS, CAT, CDASI and Skin VAS) were all completed and recorded by trained pediatric rheumatologists and trainees, and/or an advanced practice specialty clinician at each visit as part of routine clinical care. The clinic lead (BMF) trained the other clinicians in scoring the tools; he had worked on the validation studies for several of the skin scores. While specific training resources, and atlases, exist, these were variably used by the study team.

### Statistical analyses

#### Skin score correlations

The Spearman coefficient (r_s_) was used to measure the degree of correlation amongst all four skin tools at baseline, for the whole cohort. In the case of missing values, pairwise deletion was used. We considered r_s_ = 0.5 to 0.7 as moderate, and > 0.7 as high correlation. To assess the longitudinal correlations, three separate GEE models were fitted, with Physician VAS as the response being predicted by one of skinDAS, CAT, or CDASI. All GEEs used an autoregressive correlation structure. GEE is considered to be robust to missing data that is missing completely at random. These models were used to calculate the Mean Absolute Error (MAE) associated with each measurement tool over all the visits over time. These absolute errors of the DAS, CAT, and CDASI represent the standardized absolute differences between the model estimates of the Skin VAS and the observed scores. The MAEs for the three measurement tools were then visualized over time using locally weighted least squares regression (LOESS).

#### Responsiveness

In a separate analysis, for the nested inception cohort, skin scores at the first visit and after three months of treatment were extracted to assess responsiveness to change. Since we expect, on average, skin rash to improve in the first three months following therapy, we independently evaluated standardized response means (SRM) for each of the skinDAS, Skin VAS, CAT, and CDASI. Bootstrap resampling methods were used with 100 replications to derive 95% confidence intervals for the SRM values.

All statistical analyses were done using R 4.2.3 [23].

## Results

The study population (Table 1) comprised 77 total patients, of whom 25 were newly diagnosed. A total of 478 visits were analyzed over a 42-month period (June 1^st^, 2018 – December 31^st^, 2021).

**Table 1:** Demographic table of the sample study groups at the first study visit after June 1^st^, 2018 (baseline). The age of onset, sex, EULAR/ACR diagnostic certainty criteria, baseline features, treatment medications), and myositis specific / associated autoantibodies (MSA/MAA) are included for both the whole group (prevalence) & separately for the sub-group of the incidence cohort. One subject had both antiRo52 and anti-Jo-1, all others had no, or only one autoantibody. Autoantibody testing was performed using an immunoblot assay

Variable	Prevalence Cohort (*N* = 77)	Nested Incidence Cohort (*N* = 25)
**Age of Onset – years**		
Mean (SD)	8.2 (4.1)	9.5 (4.1)
Range	1–16	3–16
**Sex (%)**		
Female	66.2%	66.7%
**EULAR/ACR** **Diagnostic Certainty** *- no. of patients (%)*	Definite – 75 (97%) Probable – 2 (3%)	Definite – 24 (96%) Probable – 1 (4%)
**Diagnostic features of the “Probable Patients”**	Skin rash, positive autoantibodies, Abnormal Gottron’s papules, chest pain, respiratory discomfort, fatigue, poor sleep, reduced strength & muscle atrophy	
**Baseline Features** at the first visit after June 1^st^ 2018 **(Mean ± SD) or (%)**		
Manual Muscle Testing of 8 groups **(MMT-8, possible range 0 – 80)**	**(*N* = 32)** 72.1 ± 10.0	**(*N* = 20)** 69.4 ± 10.6
Childhood Myositis Assessment Scale (**CMAS, possible range 0 – 52)**	**(*N* = 72)** 44.9 ± 10.7	**(*N* = 24)** 39.8 ± 13.2
Childhood Health Assessment Questionnaire **(CHAQ, possible range 0 – 3)**	**(*N* = 26)** 0.7 ± 0.8	**(*N* = 17)** 1.0 ± 0.8
Disease Severity at Onset by means of the **Physician’s skin VAS (possible range 0 – 10)**	1.8 ± 2.1	3.6 ± 1.9
Skin Ulcers*	*Normal / None – 67 (87%)* *Abnormal – 9(12%)* *Missing—1*	*Normal / None – 19 (76%)* *Abnormal – 6(24%)*
Gottron’s Papules*	*Normal / None – 41 (53%)* *Abnormal – 35(45%)*	*Normal / None – 3 (12%)* *Abnormal – 22(88%)*
Heliotrope Rash*	*Normal / None – 44(57%)* *Abnormal – 32 (42%)*	*Normal / None – 6 (20%)* *Abnormal – 19 (80%)*
Nailfold Capillary*	*Normal / None – 30(39%)* *Abnormal – 46 (60%)*	*Normal / None – 4 (16%)* *Abnormal – 21 (84%)*
**Treatment Medications** *- no. of patients (%) on Tx at first visit after June 1, 2018*		
Prednisone	23 (30%)	15 (60%)
Methotrexate	37 (48%)	14 (56%)
IVIG	11 (14%)	2 (8%)
Hydroxychloroquine	5 (6%)	1 (4%)
Cyclophosphamide	1 (1%)	1 (4%)
Cyclosporin	0 (0%)	0 (0%)
MMF	0 (0%)	0 (0%)
**Myositis Specific / Associated Autoantibodies (MSA/MAA)** *– no. of patients at any time (%)*	Number tested = 40	Number tested = 22
Extractable nuclear antigen **(Anti-Ro52/SSA)**	6 (15%)	4 (18%)
Melanoma Differentiation- Associated gene 5 (**Anti-MDA-5**)	2 (5%)	1 (5%)
Transcription Intermediary Factor 1(**Anti-TIF-1**)	1 (3%)	1 (5%)
Anti-histidyl transfer RNA [t-RNA] synthetase (**Anti-Jo-1**)	1 (3%)	1 (5%)
Nucleosome Deacetylase Complex; helicase binding protein (**Anti-Mi-2**)	0 (0%)	0 (0%)
Nuclear matrix protein 2 (**Anti-NXP2)**	0 (0%)	0 (0%)
All negative	30 (75%)	15 (68%)

^*^Assessed separately from the skin scores in the clinical pro forma. Nailfold capillary abnormalities were determined by handheld microscopy in most patients, with some patients undergoing video microscopy as well

At baseline, 4 CAT scores (1 incidence cohort, 3 prevalence cohort), 1 skinDAS score (prevalence), 6 Skin VAS scores (3 incidence and 3 prevalence), and 5 CDASI scores (1 incidence, 4 prevalence) were missing.

The median (25%ile, 75%ile, range of values) CAT score was 3 (0, 7, 0–66) out of a maximum possible activity score of 96, skinDAS was 4 (1, 6, 0–9) out of a maximum score of 9, CDASI 2 (0, 6, 0–55) out of a maximum possible activity score of 100, and Skin VAS was 1 (0, 2.5, 0–9) out of a maximum possible score of 10.

In the whole cohort, all the scores demonstrated large intercorrelations [24] at the baseline assessment (0.79 – 0.92) (Table 2). While the skinDAS had the lowest correlation with the Skin VAS at baseline, the differences were very small and likely unimportant.

**Table 2:** Baseline Spearman Correlation (r_s)_ matrix for the Cutaneous Assessment Tool (CAT), skin portion of the Disease Activity Score (skinDAS), Cutaneous Dermatomyositis Disease Area and Severity Index (CDASI) & Physician’s skin overall activity visual analog scale (VAS)

Correlation Coefficient Values at Baseline				
	**CAT Total Score**	**skinDAS Total Score**	**CDASI Total Score**	**Physician's skin VAS Score**
**CAT Total Score**	1			
**skinDAS Total Score**	0.86	1		
**CDASI Total Score**	0.92	0.87	1	
**Physician’s skin** **VAS Score**	0.86	0.79	0.83	1

All *p* < 0.001

**Fig. 1:**
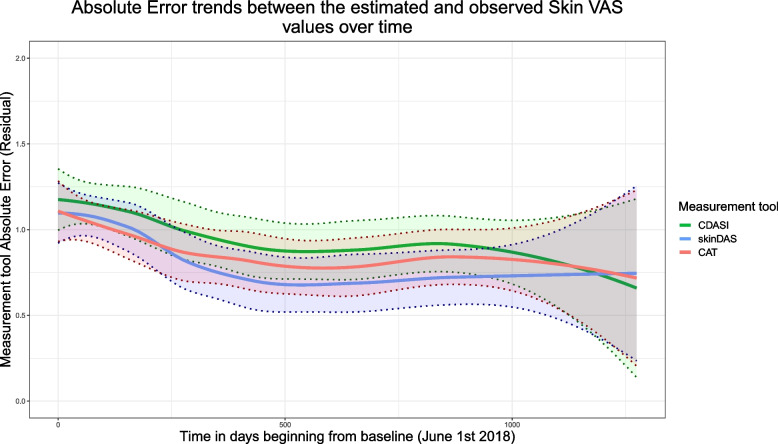
Locally weighted smoothing plotting differences in Mean Absolute error (i.e., difference between the plotted skin score and the Skin VAS) over time in days since baseline (since June 1^st^, 2018). 95% Confidence Intervals have been added for the CDASI, skinDAS, and CAT (shaded). The confidence intervals are largely overlapping as demonstrated by the overlapping colors

Standardized Mean Absolute Error (SMAE) values for the CAT, skinDAS and CDASI are presented in Table 3. All values ranged from 0.89–1.02. While the skinDAS had the lowest SMAE, all were strongly, and similarly, related over time with the Skin VAS. That is, all the measures closely paralleled the Skin VAS as it changed over time. Figure 1 illustrates this relationship over time using a smoothed curve for each measurement tool.

**Table 3:** skinDAS, CAT & CDASI Mean Absolute Error (MAE) over all N observations (478)

Measurement Tool	Standardized Mean Absolute Error (95% CI)
Skin Disease Activity Score (skinDAS)	0.89 (0.81–0.97)
Cutaneous Assessment Tool (CAT)	0.93 (0.85—1.01)
Cutaneous Dermatomyositis Disease Area and Severity Index (CDASI)	1.02 (0.94—1.10)

Standardized MAE values were calculated for each measurement tool by taking the sum of the absolute value of the differences between the GEE model’s predictions of the global Physician VAS and the observed values and dividing them by the total amount of observations (478 observed values), 95% confidence intervals are included

**Fig. 2:**
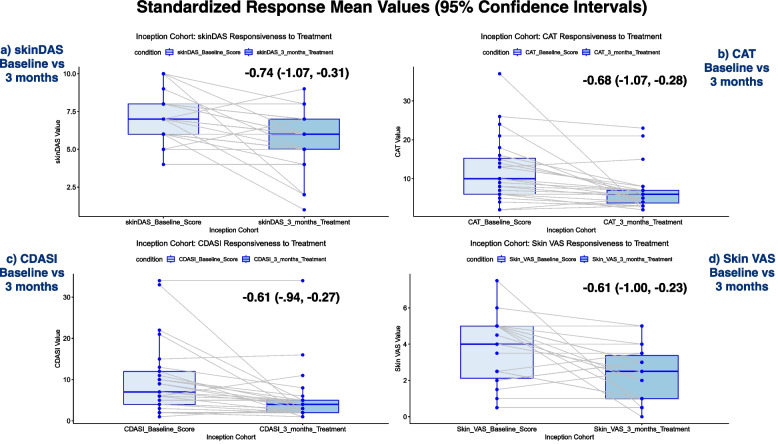
Box plots at diagnosis and 3 months later in the inception cohort. Standardized Response Mean (SRM) values are listed with 95% Confidence Intervals for the a) skinDAS, b) CAT, c) CDASI and d) Skin VAS. SRM values are calculated by the amount in change of the score, standardized by the standard deviation of that change – so that they are all valued in the same units, even though the questionnaires have different levels of scoring. That is, they are measures of standard deviations of change (with more being better in patients that are expected to have changed)

Skin score responsiveness to treatment, in the nested inception cohort, was moderate-to-large for all tools (Fig. 2). The highest SRM was seen with the skinDAS (–0.74, meaning that the score improved by 0.74 standard deviations of the change in the score), and the lowest was for the CDASI and the Skin VAS (SRM = 0.61); however, the confidence intervals were widely overlapping suggesting no evidence to support one score as being more responsive than the others.

## Discussion

We found that the skinDAS, CAT and CDASI all behave similarly; they correlate closely and closely track the Skin VAS score over time and are moderately to highly responsive in demonstrating improvement with therapy. The skinDAS has fewer items, and is therefore somewhat simpler to score, is marginally more responsive, but has a slightly weaker association with the Skin VAS; however, there were no statistically significant advantages for any one tool. The skin score tools vary in their degree of complexity and detail. For widespread comparative care, and for research, picking one of these scores to act as the standard will involve arbitrary consensus as none far outperforms the others.

As it is our clinical practice to do a complete skin evaluation for every clinic visit, we cannot comment on the difference in time taken to complete the scores; for us, the only difference in time was due to the time taken to document and score the tools. As we consider it to be best practice to do a complete skin evaluation, there is probably little difference between the scores in terms of feasibility in the clinic.

Our results must be interpreted in the light of several possible limitations. Given the rarity of the condition, and the sample numbers available, the skin scores’ relationship over time was examined without accounting for the effects of potential modifying factors like age, sex, gender, treatment type etc. By including these variables in the statistical analyses, new interactions and measurement tool relationships might be understood. The relatively small sample size of inception patients will have reduced the precision of the responsiveness statistics; however, the estimates are all very similar and we do not expect that a lack of precision has hidden an important difference amongst the tools. It should be noted that some tools may be more responsive in highly active patients, and some in patients with low disease activity; our limited sample size did not allow for stratification by starting scores. Another potential limitation is missing data amongst measurement tools. During visits, each of the four tools were expected to be used; however, there was some missing data that appeared to be missing completely at random. (Values were missing due clinician being too busy while charting during crowded clinics, and not due to characteristics of the patient.) Although standard GEE models are valid under the assumption of missing completely at random (MCAR), and robust to missingness at random, bias can be introduced if this is not the case. Additionally, different physicians (with different levels of experience) and an advanced practice clinician completed the assessments at different visits. The lack of randomization of the order of clinicians completing these scores may have affected their scoring procedure. Clinicians who routinely recorded the same tool first may carry over their scoring and judgement to the other tools, impacting the remaining skin scores (and thus leading to the high correlations). Future research should likely randomize the order of scoring when comparing skin tools. It has been suggested that rheumatologists may be more or less subjective when categorizing skin disease based on the experience of the rater [12]. This may have affected the measurement performance of the skin scores as compared to the Skin VAS.

It should be born in mind that the skin tools were developed for different purposes. Though all 4 tools assess skin in JDM, they are varied in their detail. There may be value (e.g., for research or registries) in quantitative assessment in specific skin findings (see above) as each individual with JDM likely has different skin disease, and different aspects of skin disease may respond differently (and have differential impact for the patient or clinician) in longitudinal assessment. While overall skin disease assessment may correlate, assessment of specific skin disease aspects and their response will likely be differentially lost with some skin tools.

## Conclusions

Muscle disease is often the dominant clinical feature in the current core set criteria for JDM. However, it has been accepted that cutaneous involvement is an equally important manifestation of JDM disease and an indication to consider more aggressive therapy [7, 25]. Skin rash is a hallmark of JDM, and is associated with poorer outcomes and poorer patient quality of life [26]. Furthermore, studies have emphasized the importance of residual skin change in JDM patients and the association that, for example, persistent capillary abnormalities have with extended disease course [27].

Measuring the activity of skin disease in children with JDM is important; having a single scoring tool to act as a global standard would be desirable. We have shown that the currently widely recommended skin scoring tools have similar measurement properties for overall skin assessment. A decision about which skin score should serve as the global standard will, therefore, depend on arbitrary consensus.

## Data Availability

The datasets generated during and/or analysed during the current study are not publicly available due to privacy and confidentiality reasons.

## Abbreviations

ACR: American College of Rheumatology
CAT: Cutaneous Assessment Tool
CDASI: Cutaneous Dermatomyositis Disease Area and Severity Index
CHAQ: Childhood Health Assessment Questionnaire
CMAS: Childhood Myositis Assessment Scale
EULAR: European League Against Rheumatism
GEEs: Generalized estimating equations
IMACS: The International Myositis Outcome Assessment Collaborative Study Group
JDM: Juvenile Dermatomyositis
LOESS: Locally weighted least squares regression
MAE: Mean Absolute Error
MCAR: Missing completely at random
MMT-8: Manual Muscle Testing Subset of Eight Muscles
MSA: Myositis-Specific Antibodies
PRINTO: The Paediatric Rheumatology International Trials Organization
REB: Research Ethics Board
skinDAS: Disease Activity Score
Skin VAS: Physician’s global assessment skin visual analog scale
SRM: Standardized response mean
